# Dolabelladienols A–C, New Diterpenes Isolated from Brazilian Brown Alga *Dictyota pfaffii*

**DOI:** 10.3390/md12074247

**Published:** 2014-07-23

**Authors:** Alonso Pardo-Vargas, Ingrid de Barcelos Oliveira, Paulo Roberto Soares Stephens, Claudio Cesar Cirne-Santos, Izabel Christina Nunes de Palmer Paixão, Freddy Alejandro Ramos, Carlos Jiménez, Jaime Rodríguez, Jackson Antonio Lamounier Camargos Resende, Valeria Laneuville Teixeira, Leonardo Castellanos

**Affiliations:** 1 Departamento de Química, Universidad Nacional de Colombia, Cra. 30 N° 45-03, Bogotá D.C., Colombia; E-Mails: apardov@unal.edu.co (A.P.-V.); faramosr@unal.edu.co (F.A.R.); 2 Laboratório de Imunologia Clínica, Departamento de Imunologia, Instituto Oswaldo Cruz/FIOCRUZ, Av. Brasil 4365, Manguinhos, Pavilhão Leônidas Deane/409, Rio de Janeiro 21045-900, RJ, Brazil; E-Mails: ingridbarcelos@gmail.com (I.B.O.); paulo2000@yahoo.com.br (P.R.S.S.); claudiocirne@gmail.com (C.C.C.-S.); 3 Laboratório de Virologia Molecular e Biotecnologia Marinha, Programa de Pós-graduação em Ciências e Biotecnologia, Departamento de Biologia Celular e Molecular, Instituto de Biologia, Universidade Federal Fluminense, Niterói 24020-141, RJ, Brazil; E-Mail: izabeluff@gmail.com; 4 Departamento de Química Fundamental, Facultade de Ciencias and Centro de Investigaciones Científicas Avanzadas (CICA) Universidade da Coruña, 15071 A Coruña, Spain; E-Mails: carlos.jimenez@udc.es (C.J.); jaime.rodriguez@udc.es (J.R.); 5 Laboratório de Difração de Raios X, Instituto de Química, Universidade Federal Fluminense, Niterói 24210-346, RJ, Brazil; E-Mail: jresende@id.uff.br; 6 Laboratório Algamar, Departmento de Biologia Marinha, Instituto de Biologia, Universidade Federal Fluminense, Niterói 24020-141, RJ, Brazil

**Keywords:** marine natural products, *Dictyota pfaffii*, dolabellane diterpenes, anti-HIV-1

## Abstract

The marine brown alga *Dictyota pfaffii* from Atol das Rocas, in Northeast Brazil is a rich source of dolabellane diterpene, which has the potential to be used in future antiviral drugs by inhibiting reverse transcriptase (RT) of HIV-1. Reexamination of the minor diterpene constituents yielded three new dolabellane diterpenes, (1R*,2*E*,4R*,7*S*,10S*,11S*,12R*)10,18-diacetoxy-7-hydroxy-2,8(17)-dolabelladiene (**1**), (1R*,2*E*,4R*,7R*,10S*,11S*,12R*)10,18-diacetoxy-7-hydroxy-2,8(17)-dolabelladiene (**2**), (1R*,2*E*,4R*,8*E*,10S*,11*S*,12R*)10,18-diacetoxy-7-hydroxy-2,8-dolabelladiene (**3**), termed dolabelladienols A–C (**1**–**3**) respectively, in addition to the known dolabellane diterpenes (**4**–**6**). The elucidation of the compounds **1**–**3** was assigned by 1D and 2D NMR, MS, optical rotation and molecular modeling, along with the relative configuration of compound **4** and the absolute configuration of **5** by X-ray diffraction. The potent anti-HIV-1 activities displayed by compounds **1** and **2** (IC_50_ = 2.9 and 4.1 μM), which were more active than even the known dolabelladienetriol **4**, and the low cytotoxic activity against MT-2 lymphocyte tumor cells indicated that these compounds are promising anti-HIV-1 agents.

## 1. Introduction

Human immunodeficiency virus (HIV) remains a cause of worldwide concern. According to the WHO and UNAIDS in October 2013, approximately 35.3 million people were infected with HIV. In 2012, approximately 1.6 million people died of AIDS-related causes. Since the introduction of the highly active anti-retroviral therapy (HAART), the life expectancy of HIV-infected individuals has increased, even in cases in which the virus remains active [1]. However, the discovery and development of new drugs candidates is required, as current drugs do not completely eradicate HIV from infected tissues and the long-term use of these drugs is restricted by the emergence of drug-resistant viruses, metabolic disorders and toxicity [1,2]. Of the several classes of anti-HIV compounds, inhibitors of reverse transcriptase are considered one of the most successful compounds in clinical use. Research has proven marine organisms to be excellent sources of biologically active compounds with the potential to combat HIV [1]. In particular, terpenoids isolated from marine algae [1,3,4] have been described as anti-HIV-1 inhibitors.

**Figure 1:**
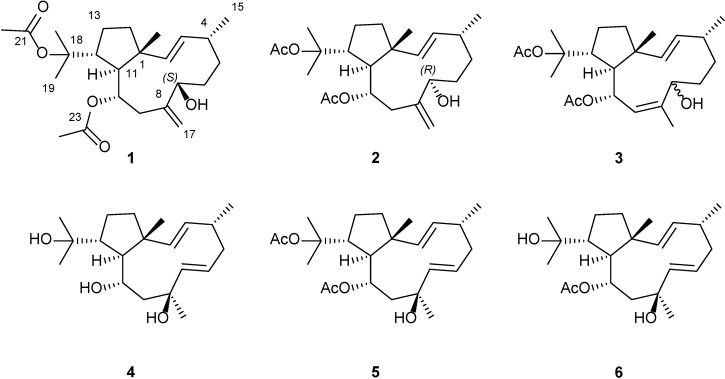
Structure of the new dolabellane diterpenoids dolabelladienols A–C (**1**–**3**), along with the known dolabelladienetriol (**4**–**6**) isolated from *D. pfaffii*.

Mechanisms of action include the blocking of different steps of the HIV-1 replicative cycle as entry inhibitors [5,6], reverse transcriptase inhibitors [1,5] or protease inhibitors [1,7]. More particularly, diterpenes isolated from marine alga represent one of the most active and promising compounds in the development of new drugs to control HIV-1 during the infective process [1,8]. In our search for new anti-HIV compounds from marine sources, we have identified a dolabellane diterpene from the brown seaweed *Dictyota pfaffii* Schnetter (Dictyotaceae, Phaeophyceae) with strong anti-HIV-1 activity. Due to the highly relevant results obtained thus far, we further investigated the anti-HIV inhibiting capacities of the minor dolabellane diterpenes. Moreover, dolabellane diterpenes demonstrate a wide array of interesting pharmacological properties such as antibacterial [9], antiprotozoal [10], antifungal [11], anti-inflammatory [12] and antimalarial [13] activities. We found one such dolabellane diterpene, dolabelladienetriol, to be a non-competitive inhibitor of HIV-1 reverse transcriptase [2,14,15]. In this paper we report the isolation and structural determination of three new dolabellane diterpenes **1**–**3**, (Figure 1), as will be demonstrated, two of these compound, **1** and **2**, exhibited potent antiviral activity.

## 2. Results and Discussion

Specimens of *D. pfaffii* were collected during scuba dives (6–9 m) at Atol das Rocas reef, Rio Grande do Norte State, the only atol in the South Atlantic, and were subsequently air-dried. In order to obtain 3.5 g of organic residue, the material was extracted exhaustively with CH_2_Cl_2_, filtered and solvent removed under vacuum at 40 °C. The resulting extract was fractionated by vacuum column chromatography (VC) and subjected to successive silica gel column chromatographies (CC) in order to afford pure compounds **1**–**6**.

### 2.1. Structural Elucidation

**Figure 2:**
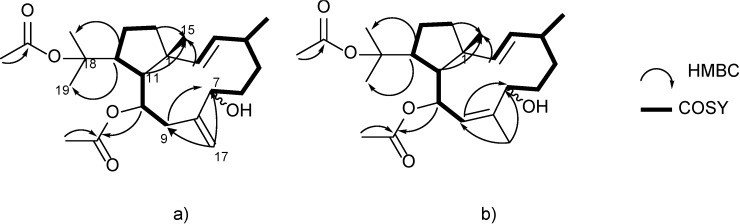
^1^H-^1^H COSY correlations (bold lines) and key H→C HMBC correlations (arrows) for compounds (**a**) **1**–**2** and (**b**) **3**.

Compound **1** (28 mg) was isolated as a white solid. EIMS analysis (*m*/*z* 406 [M]^+^) and ^13^C NMR spectra, which was confirmed by the observation of a [M + Na]^+^ peak at *m*/*z* 429.2631 in its (+)-HRESIMS suggested the molecular formula of C_24_H_38_O_5_ (six degrees of unsaturation) for compound **1**. The ^13^C NMR data of **1** (Table 1) showed the presence of 24 carbon signals, which were assigned, using an APT experiment, as six methyls, six methylenes (five sp^3^ and one sp^2^), seven methines (two sp^3^ and five sp^2^) and five quaternary carbons (three sp^3^ and two sp^2^). A detailed analysis of 1D and 2D NMR spectra determined the gross structure of **1** (Supplementary Figures S1–S6). The presence of two acetate residues were easily deduced from the ester carbonyl signals at δ_C_ 170.5 and 170.1, along with two methyl groups at δ_H_ 2.04 (s, 3H) and 1.93 (s, 3H). Therefore, we inferred the diterpene structure of **1** from the 20 carbons after subtraction of the four carbons associated with two acetate groups. The presence of two oxygenated methines were deduced from NMR signals at δ_C_ 70.2 which was coupled via HSQC with the proton signal at δ_H_ 4.37 (dd, *J* = 8.4, 3.5 Hz) and at δ_C_ 68.6 correlated with the proton signal at δ_H_ 4.91 (ddd, *J* = 8.9, 4.7, 2.4 Hz) which was assigned to an hydroxymethine and an acetoxy-bearing methine, respectively. The methlylene carbon NMR signal observed at δ_C_ 113.4 which correlated according to HMBC with the olefinic methylene protons at δ_H_ 5.12 (1H, brs) and 4.99 (1H, brs), along with olefinic quaternary carbon at δ_C_ 146.2, indicated the existence of an exocyclic double bond. The existence of disubstituted double bond with an *E* configuration in **1** was inferred by the proton signals at δ_H_ 5.66 (dd, *J* = 16.4, 7.2 Hz) and 5.17 (d, *J* = 16.4 Hz), which correlated according to HMBC with the sp^2^ carbons at δ_C_ 133.6 and 137.5. The quaternary signal at δ_C_ 84.8, characteristic of a bearing acetoxy quaternary carbon, along with the methyl singlets at δ_H_ 1.60 (s, 3H), 1.42 (s, 3H) were indicative of the presence of a 2-propan-2-yl acetate group [15]. Furthermore, the NMR data of **1** (Table 1) displayed an additional two methyl groups at δ_H_ 0.97 (d, *J* = 6.9 Hz, 3H)/δ_C_ 22.2 and δ_H_ 0.83 (s, 3H)/δ_C_ 16.8. The above-presented NMR data closely resemble those for bicyclic dolabellane skeleton, which were established as taxonomic markers for this alga [16]. However, differences in the chemical shifts as a result of the unsaturation and oxygenation pattern indicated of a new natural product. Two main proton spin systems, outlined in Figure 2a, were established by ^1^H-^1^H COSY experiments after assignments of the direct ^1^H-^13^C correlations via HSQC analysis.

The first spin system was identified by ^1^H-^1^H COSY correlations between the olefinic proton at δ_H_ 5.17 (H-2) and the hydroxymethyne at δ_H_ 4.37, (H-7). A second was recognized by correlating the methylene protons at δ_H_ 2.43 and 2.46 (H-9) with those at δ_H_ 1.46 and 1.39 (H-14). Analysis of the HMBC spectrum granted the connectivity of these two spin-systems. The observed correlations between the methylene olefinic protons H-17a (δ_H_ 5.12, brs) and H-17b (δ_H_ 4.99, brs) and the oxygenated methine C-7 (δc 70.2) and methylene C-9 (δc 41.0) allowed for the connection of the exocyclic double bond Δ8(17) to both spin systems. On the other hand, HMBC cross-peaks between the olefinic H-2 (δ_H_ 5.17, d, *J* = 16.4 Hz), the methylene H_2_-14 (δ_H_ 1.46, m) and H-11 (δ_H_ 1.84, dd, *J* = 10.8, 2.4 Hz) protons with the methyl carbon C-15 (δ_C_ 16.8) confirmed the bicyclic dolabelladiene structure presented in Figure 2a. The location of the acetate group at C-10 was established through the HMBC correlations between H-10 (δ_H_ 4.91, ddd, *J* = 8.9, 4.7, 2.4 Hz) and H_3_-24 (δ_H_ 1.93, s) protons and the carbonyl signal at δ_C_ 170.1 (C-23).

**Figure 3:**
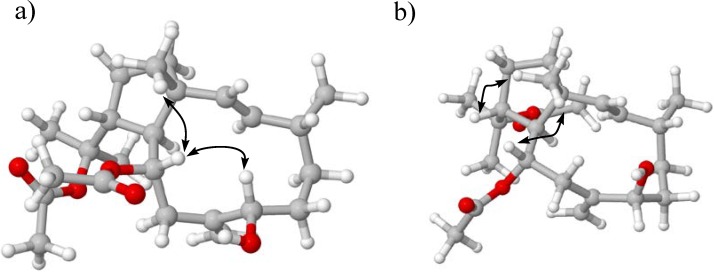
Key NOE Correlations for the main conformers found for compounds **1** (**a**) and **2** (**b**).

We demonstrated the assignment of the 2-propan-2-yl acetate group to position 12 by was the long range ^1^H-^13^C coupling of the methine proton H-12 at 2.95 (td, 10.8, 4.7) with methyl singlets C-18 and C-20 at δ_C_ 23.4 and 26.4 (Figure 2a). Therefore, on the basis of the above analysis, we established the planar structure of **1** as illustrated in Figure 2a. Having established the planar structure of dolabellane **1**, we focused our attention on its stereochemistry. Key NOESY correlations observed between methyl protons H_3_-15, H-12 and H-10, and this in turn to H-7, led to the conclusion that protons H_3_-15, H-12, H-10 and H-7 are on the same side of the bicyclic ring (Figure 3a) and further suggested a 1R*, 7S*, 10S* and 12R* relative configuration. In order to confirm these stereochemical assignments, a conformational space of the two possible 7R*- or 7S*-epimers was explored using the GMMX stochastic conformational search procedure as implemented in the program PC-MODEL, which itself used an MMX force field.

**Table 1:** ^13^C and ^1^H NMR data (500 MHz in CDCl_3_) of dolabelladienols A–C (1–3) (δ in ppm, *J* in Hz).

Position	1		2		3	
	δ_C_	δ_H_	δ_C_	δ_H_	δ_C_	δ_H_
1	48.4	-	48.1	-	48.9	-
2	137.5	5.17 (d, 16.4)	138.9	5.24 (d, 16.4)	136.9	5.23 (dd, 16.4, 0.7)
3	133.6	5.66 (dd, 16.4, 7.2)	134.5	5.53 (dd, 16.4, 8.5)	134.1	5.70 (dd, 16.4, 6.9)
4	34.8	2.20 (m)	34.7	2.04 (m)	34.9	2.39 (ddd, 13.9, 7.7, 3.3)
5	32.0	1.58 (m) 1.63 (m)	33.2	1.76 (dd, 11.2, 2.3) 1.08 (dd, 11.2, 4.9)	33.5	2.34 (m) 1.71 (m)
6	32.6	1.62 (m) 1.90 (m)	33.3	1.85 (dd, 9.0, 4.9) 1.64 (m)	30.7	1.45 (m) 1.66 (m)
7	70.2	4.37 (dd, 8.4, 3.5)	76.7	3.95 (dd, 11.4, 4.9)	69.0	5.08 (t, 6.6)
8	146.2	-	144.8	-	140.1	-
9	41.0	2.43 (dd, 13.2, 4.7) 2.46 (dd, 13.2, 8.9)	36.5	2.65 (dd, 13.8, 10.1) 2.50 (br d, 13.8)	125.8	5.31 (d, 10.4)
10	68.6	4.91 (ddd, 8.9, 4.7, 2.4)	70.4	4.88 (ddd, 10.1, 3.2, 2.1)	67.9	5.58 (dd, 10.4, 2.3)
11	51.1	1.84 (dd, 10.8, 2.4)	50.6	1.94 (dd, 10.5, 3.2)	53.3	1.76 (dd, 10.1, 2.1)
12	44.2	2.95 (td, 10.8, 4.7)	44.2	3.06 (td, 10.5, 4.9)	44.3	2.98 (m)
13	26.4	1.95 (m) 1.45 (m)	26.5	1.96 (m) 1.48 (m)	26.2	1.95 (m), 1.46 (m)
14	38.9	1.46 (m) 1.39 (m)	38.7	1.51 (m) 1.47 (m)	39.2	1.49 (m) 1.39 (m)
15	16.8	0.83 (s)	18.4	0.83 (s)	16.9	0.93 (s)
16	22.2	0.97 (d 6.9 )	21.1	1.00 (d, 6.7)	19.8	0.96 (d, 6.9)
17	113.5	5.12 (brs) 4.99 (brs)	117.0	5.04 (brs) 4.89 (brs)	17.0	1.66 (d, 1.4)
18	84.8	-	84.8	-	84.9	-
19	23.4	1.42 (s)	23.2	1.45 (s)	26.0	1.59 (s)
20	26.4	1.60 (s)	26.6	1.61 (s)	23.6	1.43 (s)
21	170.5	-	171.5	-	170.2	-
22	21.0	2.04 (s)	21.0	2.05 (s)	23.0	1.97 (s)
23	170.1	-	170.0	-	170.8	-
24	23.0	1.93 (s)	22.9	1.92 (s)	21.1	2.02 (s)

In the case of the epimers 7S* and 7R*, 37 and 23 different conformers were observed, respectively, within an energy cutoff of 3 kcal/mol. The conformations identified for the 7S* configuration alone were enough to placed protons H-7 and H-10 in a 2 Å environment, which fits the observed NOEs for the proposed stereochemistry. The 11,1-*trans*-fused ring system of the dolabellane was confirmed by the carbon chemical shift of CH_3_-15 at δ_C_ 16.8, indicating a 1R* and 11S^*^ configuration [10]. Finally, the carbon chemical shifts of C-4 methine at δ_C_ 34.8 and CH_3_-16 methyl at δ_C_ 22.2 suggested a 4R* relative configuration, which accorded with other reported compounds [15,17]. Hence, compound **1**, named dolabelladienol A, was identified as (1R*,2*E*,4R*,7R*,10S*,11S*,12R*)-10,18-diacetoxy-7-hydroxy-2,8(17)-dolabelladiene, resulting in a new natural product.

The new dolabellane diterpene **2** (6 mg) was also isolated as a white solid. Its molecular formula of C_24_H_38_O_5_ was deduced from the [M + Na]^+^ pseudo molecular ion at *m*/*z* 429.2627 (C_24_H_38_O_5_Na, ∆1.6) and ^13^C NMR spectra (Supplementary Figures S7–S12). The close resemblance between the NMR spectra of **1** and **2** (Table 1), including ^1^H-^1^H COSY and HMBC correlations (Figure 2a), indicated that **2** shares the same planar structure with **1**. However, slight differences in the NMR data of these two compounds were observed for the carbon C-7 (δ_c_ 70.2, and δ_c_ 76.7, respectively) and surrounding atoms (Table 1), which indicated that **2** corresponds to the C-7 epimer of **1**.

These findings accord with the NOESY analysis of **2** which demonstrated NOE correlations between the H_3_-15 methyl protons and H-12 and H-10 methine protons, along with the lack of correlation between H-7 and H-10 observed in the NOESY spectrum of **1** (Figure 3b). As mentioned above, the space-conformational search in a 3.0 kcal/mol range for the epimer 7*S*, showed all conformers with no dipolar correlations between H-7 and the other vicinal protons. The first is located in a populated averaged of 4.9 Å away from other proximal protons, and therefore the lack of NOE correlations in the NOESY experiment supports the hypothesis that **2** should be the epimer of **1** at C-7. Thus their stereocenters were assigned as follows 1R*,10S*,11S*,12R*. In this way, dolabelladienol A (**2**) was identified as (1R*,2*E*,4R*,7S*,10S*,11S*,12R*)10,18-diacetoxy-7-hydroxy-2,8(17)-dolabelladiene, and is here reported for the first time.

Compound **3** (4 mg) was isolated as a white solid, as well, and assigned the molecular formula C_24_H_38_O_5_ based on LREIMS analysis (*m*/*z* 406 [M]^+^) and ^13^C NMR spectra which required six degrees of unsaturation and demonstrated that **3** is an isomer of **1** (Table 1). The NMR data of compound **3** (Supplementary Figures S13–S17) also showed spectroscopic features for a dolabellane-type diterpene, similar to those of **1** and **2**, including the presence of an acetate group at C-18, an acetoxy-bearing methine at C-10 and a hydroxyl-bearing methine at C-7. By contrast, the main differences from **1** and **2** are the absence of chemical signals corresponding to the exocyclic double bond present in **1** and **2** and the presence of two endocyclic double bonds at δ_H_ 5.70 (dd *J* = 16.3, 6.9 Hz), 5.31 (d *J* = 10.4 Hz), and 5.21 (d *J* = 16.4 Hz). Assignments of H-C signals of **3** were conducted by HSQC experiments. The ^1^H-^1^H COSY experiment allowed for the establishment of two spins systems that were connected by HMBC correlations. In this way, the planar structure of compound **3** could be elucidated as shown in Figure 2b.

The relative stereochemistry of **3** could not be addressed because the compound decomposed. The *E* configuration of Δ^2^ double bond was established by the *J* = 16.4 Hz between the olefinic protons H2 and H3. Furthermore, the proton and chemical shifts were similar to the compound (1R*,2*E*,4R*,8*Z*,10S*,11S*,12R*)10,18-diacetoxydolabella-2,8-dien-6-one, reported by Lu and Faulkner in 1998, in that they share the same *Z* configuration at Δ8 double bond [18]. Comparison of the ^1^H and ^13^C NMR chemical shifts of compound **3** to those of **1** demonstrated that they share the same relative configuration for stereocenters at 1, 4, 10, 11 and 12. In this way, we identified and reported for the first time the compound **3** as (1R*,2*E*,4R*,8*E*,10S*,11S*,12R*)-10,18-diacetoxy-7-hydroxy-2,8-dolabelladiene. It is worth noting that compounds **1** or **2** may potentially convert to **3** during the course of isolation or purification due to slight acidity.

Spectral data (^1^H, ^13^C NMR, MS, optical rotation) of compound **4** (30 mg) are the same as those of dolabelladienetriol, which was previously isolated from the digestive glands of the sea hare *Dolabella californica* Stearns, 1877 [17] and obtained as semisynthetic product in 2004 [15]. It is important to note that this compound has shown strong anti-HIV and anti-HSV activity [2,19]. The dolabellanes **5** and **6** of *D. pfaffii* were also isolated and their spectral data proved identical with those of 10,18-diacetoxy-8-hydroxy-2,6-dolabelladiene (**5**) and 10-acetoxy-8,18-dihydroxy-2,6-dolabelladiene (**6**), which were previously described previously by us in 2004 [15]. We conducted a single crystal X-ray diffraction study of the compounds **4** and **5**. In the case of compound **4**, it was possible to confirm the proposed structure, but the results related to compound **5** permitted us an unambiguous description of the absolute configuration (Flack parameter = −0.03(6)). Thus, we described the configuration of compound **5** as (1*R*,2*E*,4*R*,6*E*,8*S*,10*S*,11*S*,12*R*)-8,10,18-diacetoxy-8-hydroxy-2,6-dolabelladiene.

### 2.2. Antiviral Activity

In order to establish the anti-HIV-1 potential of the isolated compounds, we first evaluated the safety of compound **1**, **2** and **4** for use in human cells by treating MT-2 lymphocytes with various concentrations of the test compound. After 4–5 days of treatment, MT-2 viability, determined colorimetrically, was higher than 90% for concentrations up to 250 μM, resulting in a CC_50_ between 1345 ± 2 and 1456 ± 3.4 μM. It is important to emphasize that the compounds were not cytotoxic and were in fact even less toxic than the neviparine (325 μM ± 1.4) used as a control.

Subsequently, we evaluated the antiviral activity of natural compounds on MT-2 cells at concentrations between 6.25 and 25 μM. At 6.25 μM, compounds **1**, **2** and **4** inhibited the HIV-1 virus in 83%, 69% and 52% of cases, resulting in EC_50_ values of 2.9 ± 0.2, 4.1 ± 0.4 and 6.16 ± 0.7 μM, respectively. There was a slight difference in activity between epimers **1** and **2**, but the antiviral activity of the 2,8(17)-dolabelladienes series was twice as active as the 2,6-dolabelladienes series and exhibited a positive influence on the inhibition of HIV-1.

It is noteworthy that when compounds **1** and **2** were evaluated in the antiviral test, they proved more active than compound **4**, hitherto the most active compound reported by us to have demonstrated non-competitive inhibition of HIV-1 reverse transcriptase [2,14,15], This suggests that these diterpenes could be considered potential new agents for HIV-1 therapy. Therefore, we recommend further studies on the precise mechanism of activity and on the *in vivo* antiviral activity.

## 3. Experimental Section

### 3.1. General Experimental Procedures

LREIMS and HRESIMS were measured on QQq-TOF mass spectrometer, Applied Biosystems QSTAR Elite. For the HRGC-MS analysis a GC7890A (Agilent Technologies, Santa Clara, CA, USA) with a DB-5MS (5% Phenyl methyl siloxane) chromatographic column (60 m × 250 μm × 0.25 μm) was coupled with an MSD-5975 (Agilent Technologies) with an electronic impact ion source (70 eV) for detecting ions between *m*/*z* 50–500 in scan mode. Injector and detector temperatures were set at 270 °C and 290 °C, respectively. We maintained the temperature program at 160 °C, and then programmed to 260 °C at a rate of 4 °C/min before finally raising the temperature at a rate of 10 °C/min to 290 °C for 15 min. Helium served as the carrier gas at a flow rate of 1 mL/min. Linear retention indices (RI) were calculated according to the Kovats method using a mixture of normal paraffin C_6_–C_26_ as external references. Nuclear magnetic resonance spectra (proton and carbon) were recorded on Varian VNMRS or Bruker 500 MHz Advance spectrometers, employing CDCl_3_ (99.8%, Merck, Whitehouse Station, NJ, USA) as a solvent. Multiplicities of ^13^C signals were obtained by DEPT. For column chromatography, we used silica gel 60. All the solvents were analytical grade. Optical rotations were measured on a Polartronic ADP440+, Bellinghan + Stanley Polarimeter (North Farm Industrial Estate, Tunbridge Wells, UK).

### 3.2. Sample Collection

The brown algae *Dictyota pfaffii* was collected during July 2009 at a depth of 6–9 m, by scuba dives at the Atol das Rocas reef, a Biological Marine Reserve in the Rio Grande do Norte State, lat. 03°51′03″S, long. 33°40′29″W, Brazil. The legal authorization for sample colleting was obtained from VLT (SISBIO/IBAMA Brazil) (number 17352). Dr. Roberto Villaça (Departamento de Biologia Marinha, Instituto de Biologia, UFF) collected and identified the seaweeds. A voucher specimen (HRJ 9117) was deposited in the herbarium of the Universidade do Estado do Rio de Janeiro (UERJ) and the seaweed was air-dried.

### 3.3. Extraction, Isolation and Structural Elucidation of Compounds

The air-dried material of *Dictyota pfaffii* (65 g) was extracted three times with 500 mL of CH_2_Cl_2_ each time for 24 h. The extracts were combined, filtered and further dried under vacuum at 40 °C. The dried crude extract (3.5 g) was subjected to vacuum column chromatography (CC) using a discontinuous gradient (Hexane/CH_2_Cl_2_/EtOAc/MeOH) in order to obtain 12 fractions (F1 to F12). Fraction F7 (CH_2_Cl_2_/EtOAc, 9:1) yields white crystals of compound **5** (750 mg). Fraction F8 was chromatographed on a silica gel CC using as eluent an isocratic mixture of CH_2_Cl_2_/EtOAc, 9:1 in order to obtain 12 fractions (F8.1 to F8.12). From these, fraction F8.3 yielded compound **2** (6 mg) and compound **3** (4 mg) after silica gel column chromatography eluted with CH_2_Cl_2_/EtOAc, 8:2 as solvent. Fraction F8.7 yielded the pure compound **6** (20 mg), and fraction F8.10 the pure compound **1** (28 mg). Finally, by crystallization in hexane, fraction F10 yielded pure compound **4** (30 mg) in the form of white needle crystals. All the isolated compounds were identified the analysis of their 1D- and 2D-NMR spectra, together with their EI-MS spectra analysis.

### 3.4. Conformational Searches

Conformational searches were performed using 50,000 GMMX steps as implemented in the PCmodel software (version 8.5), the MMFF94s force field and the TNCG algorithm. All local minima within 3.5 kcal/mol of the global minimum were saved and subsequently re-minimized using the FMNR algorithm within an energy cut-off of 3.0 kcal/mol. For compound **1**, 15 out of 37 conformers, 56.9% of the population, exhibited NOE between H-7 and H-10 (distances of 2.11 and 2.96 Å). For compound **2**, all 23 found conformers, 100% of the population, did not show NOE between H7 and H10 (distances larger than 3.8 Å).

**Dolabelladienol** **A** (1R*,2*E*,4R*,7S*,10S*,11S*,12R*)10,18-diacetoxy-7-hydroxy-2,8(17)-dolabelladiene (**1**). White solid (28 mg), 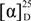 +11° (c 0.002, CHCl_3_), RI 2593. ^1^H and ^13^C NMR data, see Table 1. **EIMS** *m*/*z* (relative intensity) 406 (4), 346 (3), 304 (4), 286 (100), 271 (46), 243 (61), 215 (25), 173 (34), 145 (32), 133 (30), 107 (75), 93 (80), 55 (65). **HRESIMS**: [M + Na]^+^ 429.2631 (C_24_H_38_O_5_Na 429.2611, ∆1.95 mmu).

**Dolabelladienol** **B** (1R*,2*E*,4R*,7R*,10S*,11S*,12R*)10,18-diacetoxy-7-hydroxy-2,8(17)-dolabelladiene (**2**). White solid, 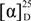 +4° (c 0.002, CHCl_3_), RI 2567. ^1^H and ^13^C NMR data, see Table 1. **HRESIMS** 835.5353 [2M + Na]^+^; 429.2627 [M + Na]^+^; **EIMS** *m*/*z* (relative intensity) 406 [M]^+^ (3), 328 [M – AcOH − H_2_O]^+^ (5), 304 (7), 286 [M − AcOH − AcOH]^+^ (45), 243 (43), 215 (20), 187 (28), 161 (47), 135 (65), 107 (77), 93 (100), 55 (60).

**Dolabelladienol** **C** (1R*,2*E*,4R*,8*E*,10S*,11S*,12R*)10,18-diacetoxy-7-hydroxy-2,8-dolabelladiene (**3**). White solid, 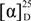 +14° (c 0.002, CHCl_3_), RI 2475. ^1^H and ^13^C NMR data, see Table 1 **EIMS** *m*/*z* (relative intensity) 406 (4) 304 (5) 286 (38) 271 (31) 243 (32) 229 (12) 203 (23) 175 (51) 133 (52) 121 (71) 107 (75) 95 (100) 79 (58) 69 (53) 55 (63).

(1R*,2*E*,4R*,6*E*,8S*,10S*,11S*,12R*)-8,10,18-trihydroxy-2,6-dolabelladiene (**4)**. White needles, 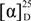 −27° (c, 0.5, CHCl_3_), RI 2257, NMR, and MS data are consistent with literature values [15].

(1*R*,2*E*,4*R*,6*E*,8*S*,10*S*,11*S*,12*R*)-10,18-diacetoxy-8-hydroxy-2,6-dolabelladiene (**5**). White needles, 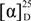 −123° (c 0.002, CHCl_3_), RI 2475, NMR, and MS data are consistent with literature values [15].

(1R*,2*E*,4R*,6*E*,8S*,10S*,11S*,12R*)-10-acetoxy-8,18-dihydroxy-2,6-dolabelladiene (**6**). White solid, 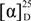 −70° (c, 0.5, CHCl_3_), RI 2314, NMR, and MS data are consistent with literature values [15].

### 3.5. X-Ray Diffraction Analysis of Compound **4**

**Figure 4:**
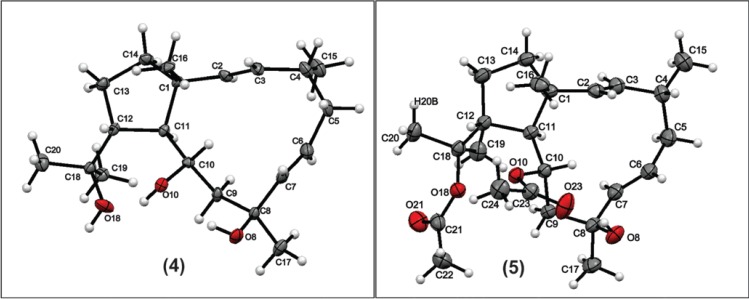
X-ray crystal structure of the compound **4** and **5**.

X-ray diffraction data was carried out in Bruker D8 Venture with radiation CuKα (λ = 1.5418 Å). The structure was solved by direct methods and refined by full-matrix least squares on F^2^ with SHELX-97 package (Figure 4) [20]. The positions of hydrogen atoms were generated geometrically and refined according to a riding model. All non-hydrogen atoms were refined anisotropically. Crystallographic data for the structures of **4** and **5** have been deposited with the Cambridge Crystallographic Data Centre database.

Crystallographic data for compound **4**: C_20_H_34_O_3_, F.W. = 322.5, colorless needle, monoclinic, space group P2_1_, a = 9.4394(7) Å, b = 7.6155(5) Å, c = 13.4810(8) Å, β = 95.527(4)°, volume = 964.59(11) Å^3^, Z = 2, Dc = 1.11g/cm^3^, μ = 0.56 mm^−1^, F(000) = 356, Crystal dimensions: 0.065 mm × 0.097 mm × 0.268 mm. Independent reflections: 3415 (Rint = 0.058). The final anisotropic full-matrix least-squares refinement on F^2^ with 216 variables converged at R1 = 9.73%, for the observed data and wR2 = 25, 38% for all data. CCDC number: 1000466.

Crystallographic data for compound **5**: C_24_H_38_O_5_, F.W. = 408.5, colorless prismatic, orthorhombic, space group P2_1_2_1_2_1_, a = 8.3069(2) Å, b = 13.6440(3) Å, c = 20.7705(5) Å, volume = 2354.11(10) Å^3^, Z = 4, Dc = 1.15 g/cm^3^, μ = 0.63 mm^−1^, F(000) = 888, Crystal dimensions: 0.16 mm × 0.29 mm × 0.50 mm. Independent reflections: 4221 (Rint = 0.041). The final anisotropic full-matrix least-squares refinement on F^2^ with 267 variables converged at R1 = 3.77%, for the observed data and wR2 = 9.87% for all data. The Flack parameter was −0.03 (6) using 1609 quotients [(I+) − (I−)]/[(I+) + (I−)] [21] CCDC number: 1005607.

### 3.6. Cells and Viruses

MT-2 cells were grown in RPMI 1640 media (LGC Bio, São Paulo, Brazil), which contained 10% heat-inactivated fetal bovine serum (Hyclone, Logan, UT, USA). Cell cultures were incubated at 37 °C in a humidified air atmosphere containing 5% CO_2_. HIV-1 isolates IIIB (X4-tropic, subtype B, donated by Eva Maria Fenyo, University of Lund, Lund, Sweden) were prepared in PHA-activated MT-2 cells and stored at −70 °C.

### 3.7. Cytotoxicity Assays

The cytotoxicity of the compounds in triplicates was tested in MT-2 cells (2 × 10^6^ cell/100 μL/well) grown in 96-well plates for 72 h at 37 °C with a 5% CO_2_ atmosphere. Cells were incubated with culture medium containing the compounds at different concentrations. After 4 to 5 days the HIV-1, cytopathic effect was determined colorimetrically by the MTT method [22]. In brief, 20 μL of MTT Sigma Chemical Co. (St. Louis, MO, USA) (5 mg/mL) were added to wells and 4 h later the optical density was measured at 540 nm. The 50% cytotoxic concentration (CC_50_) was determinate as the concentration capable of reducing the optical density by 50% in comparison with the control. The CC_50_ was calculated by linear regression analysis of the dose-response curves generate as previously described [23].

### 3.8. Antiviral Assays

The MT-2 cells (2 × 10^6^ cell/100 μL/well) were initially exposed to viral suspensions containing 5 to 10 ng/mL of HIV-1 p24 Ag for 2 to 3 h. The cells were treated with compounds at different concentrations. After 2–3 days at 37 °C in 5% CO_2_, HIV-1 cytopathic effect was determined colorimetrically by the MTT method [22]. EC_50_ was calculated as described [23]. The positive control was neviparine 5 μM (replication viral inhibition of 99%).

## 4. Conclusions

This study identified four hitherto undescribed dolabellane diterpenes from *Dictyota pfaffii* (**1**–**4**), three of which are new (**1**–**3**), while one (**4**) is here described for the first time in this species, in addition to the major known compounds **5** and **6**. Biological assays demonstrated that none of the compounds were cytotoxic. Compounds **1** and **2** were evaluated in the antiviral test and seen to be more active than dolabelladienetriol **4** and nevirapine. Such results confirm that these diterpenes should be considered as potential new agents for HIV-1 therapy.

## Supplementary Files

Supplementary File 1 Supplementary Information (PDF, 2074 KB)
