# Supplementary material for: Dolabelladienols A–C, New Diterpenes Isolated from Brazilian Brown Alga Dictyota pfaffii
Source: Mar Drugs. 2014 Jul 23;12(7):4247–59. doi: 10.3390/md12074247 (PMC4113826; doi:10.3390/md12074247)
Supplement: Supplementary File 1 — Supplementary Information (PDF, 2074 KB) [file marinedrugs-12-04247-s001.pdf]

Supplementary Information

Figure S1. ^{1}H NMR data for compound **1** in $CDCl_3$.
Figure S2. APT NMR data for compound **1** in $CDCl_3$.
Figure S3. COSY spectra for compound **1** in $CDCl_3$.
Figure S4. HSQC spectra for compound **1** in $CDCl_3$.
Figure S5. HMBC spectra for compound **1** in $CDCl_3$.
Figure S6. NOESY spectra for compound **1** in $CDCl_3$.
Figure S7. ^{1}H NMR of compound **2** (500 MHz in $CDCl_3$).
Figure S8. NMR APT spectra for compound **2** (125 MHz in $CDCl_3$).
Figure S9. COSY spectra for compound **2** in $CDCl_3$.
Figure S10. HSQC spectra for compound **2** in $CDCl_3$.
Figure S11. HMBC spectra for compound **2** in $CDCl_3$.
Figure S12. NOESY spectra for compound **2** in $CDCl_3$.
Figure S13. ^{1}H NMR of compound **3** (500 MHz in $CDCl_3$).
Figure S14. NMR APT spectra for compound **3** (125 MHz in $CDCl_3$).
Figure S15. COSY spectra for compound **3** in $CDCl_3$.
Figure S17. HMBC spectra for compound **3** in $CDCl_3$.

Figure S1. ^{1}H NMR of compound **1** (500 MHz in $CDCl_3$).

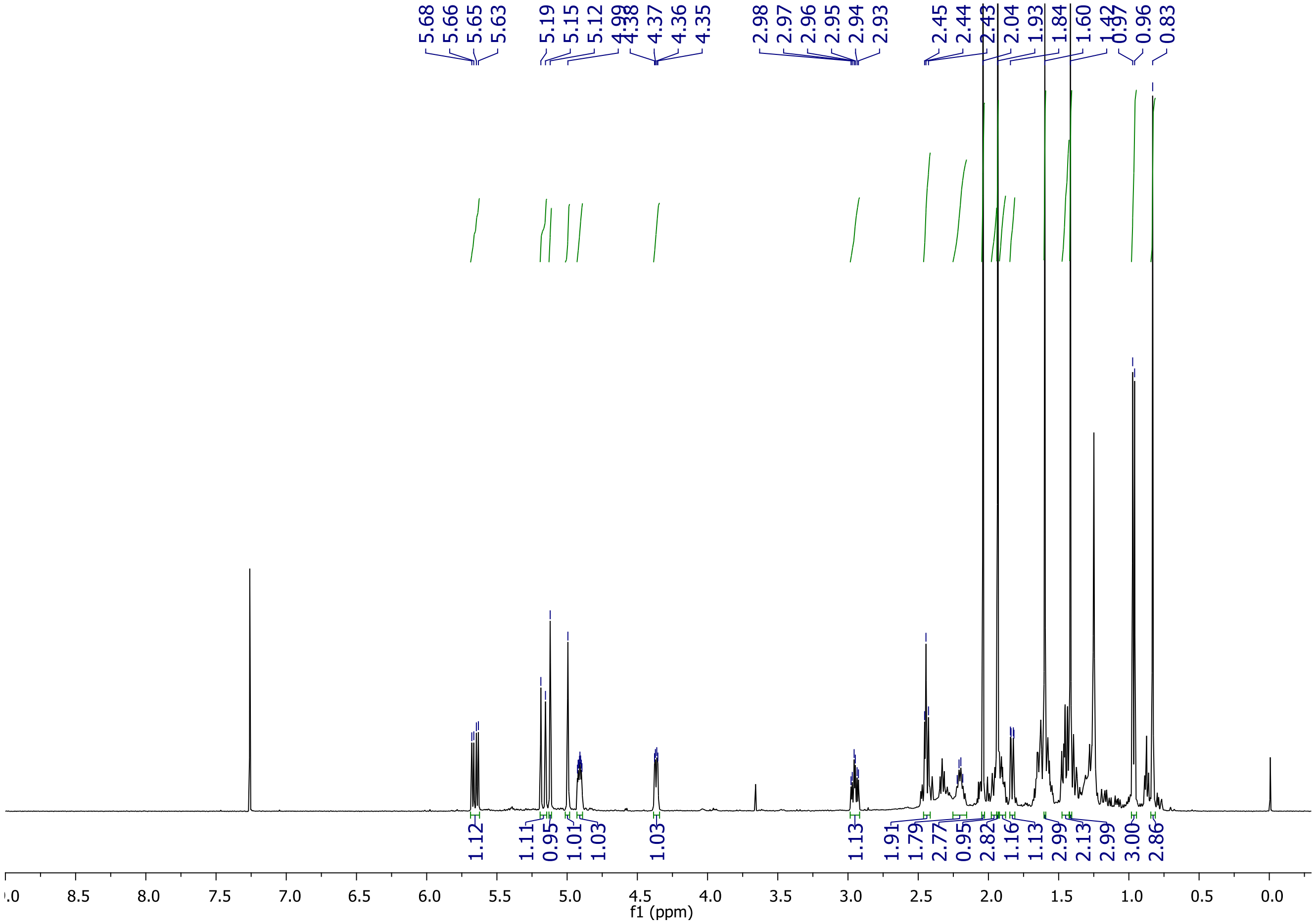

Figure S2. NMR APT spectra for compound **1** (125 MHz/CDCl$_3$).

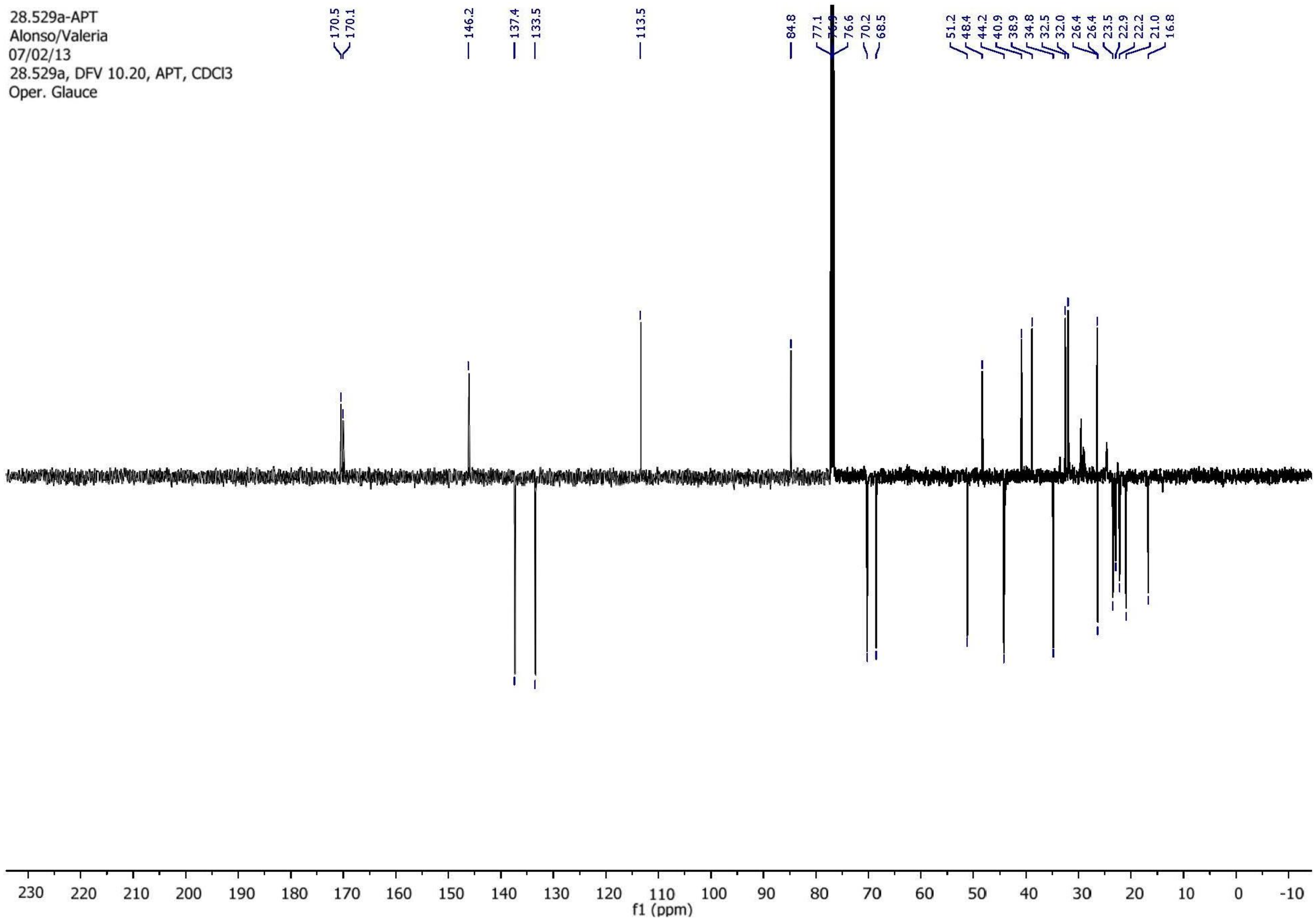

Figure S3. COSY spectra for compound **1** in $CDCl_3$.

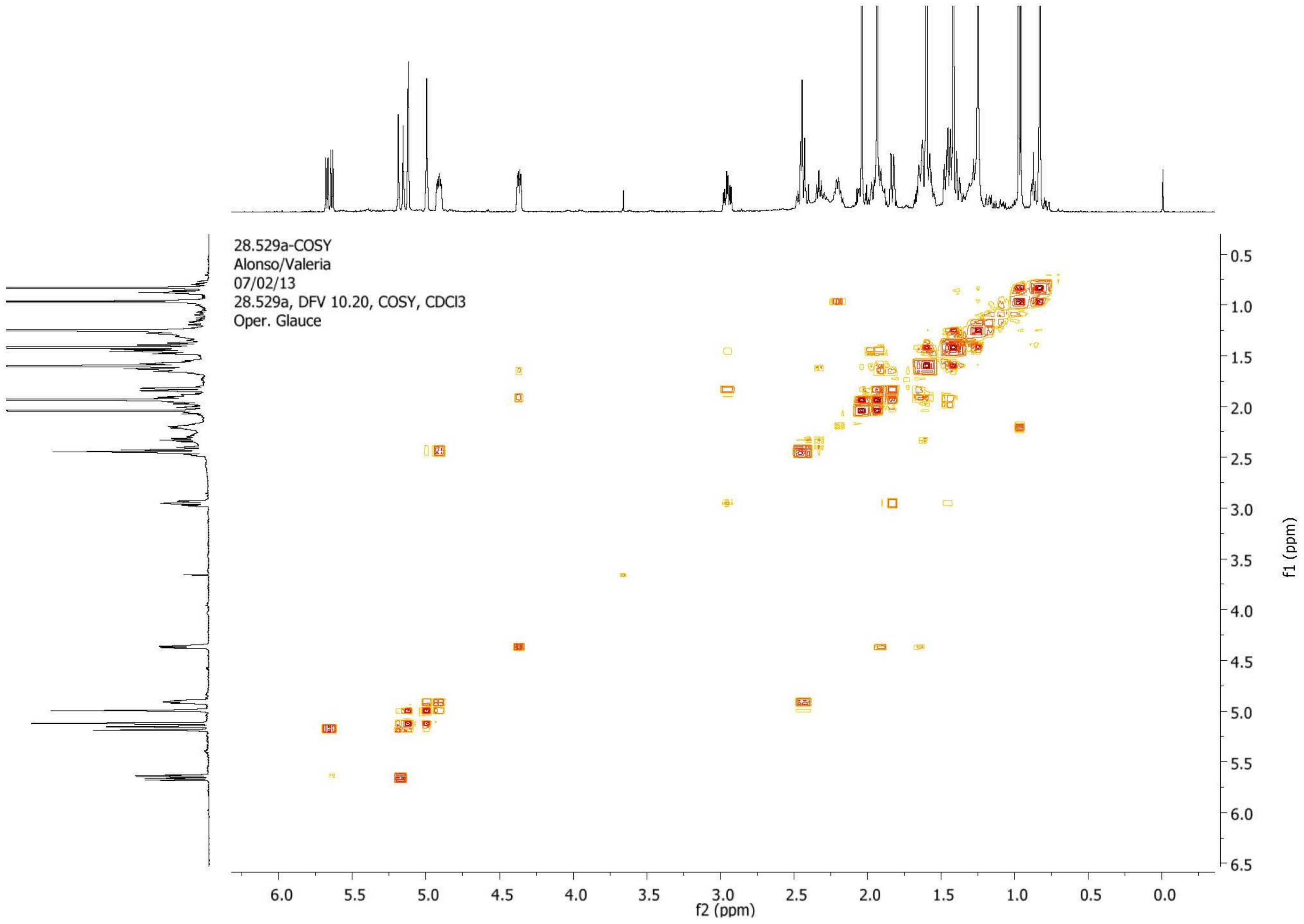

Figure S4. HSQC spectra for compound **1** in $CDCl_3$.

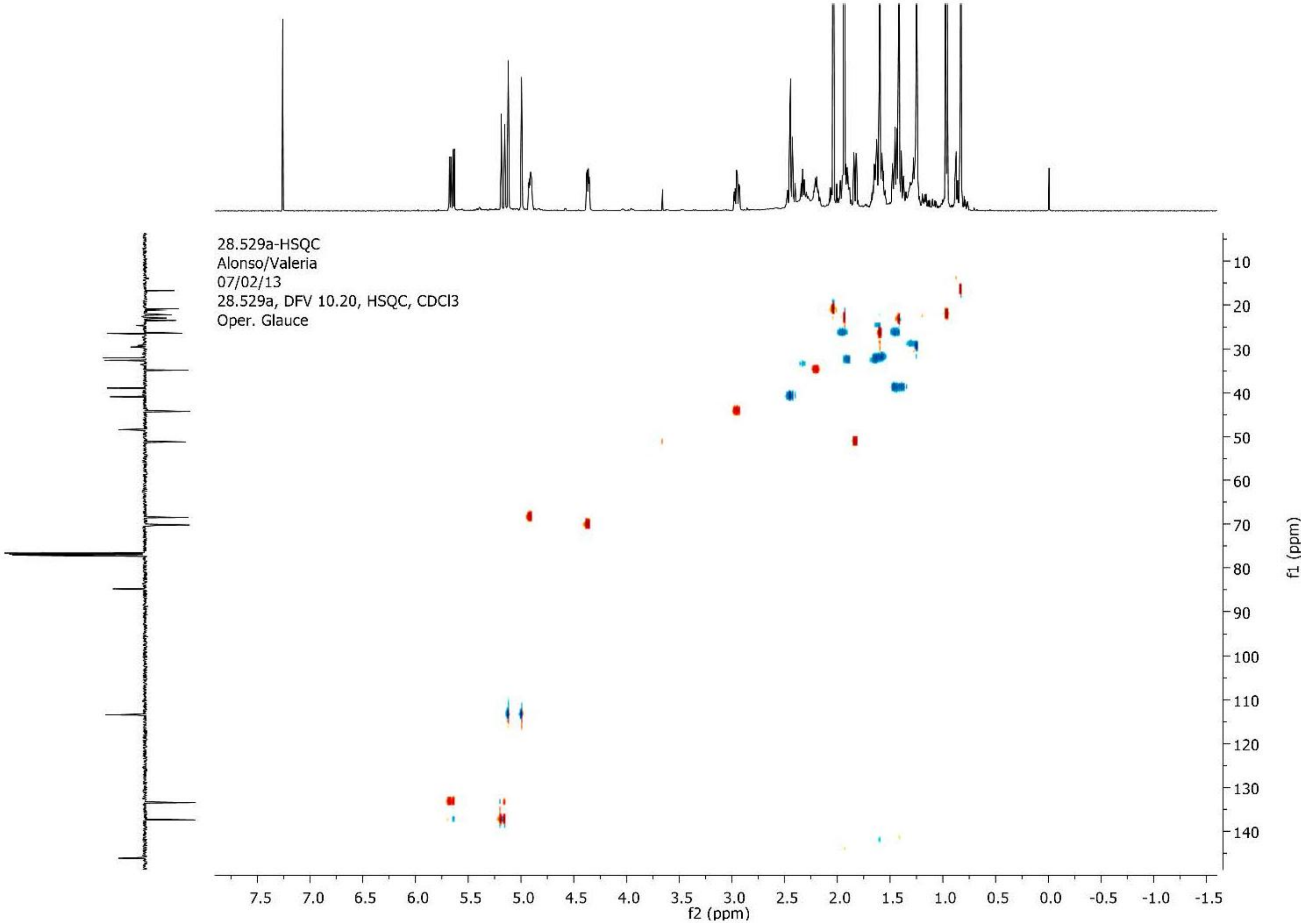

Figure S5. HMBC spectra for compound **1** in $CDCl_3$.

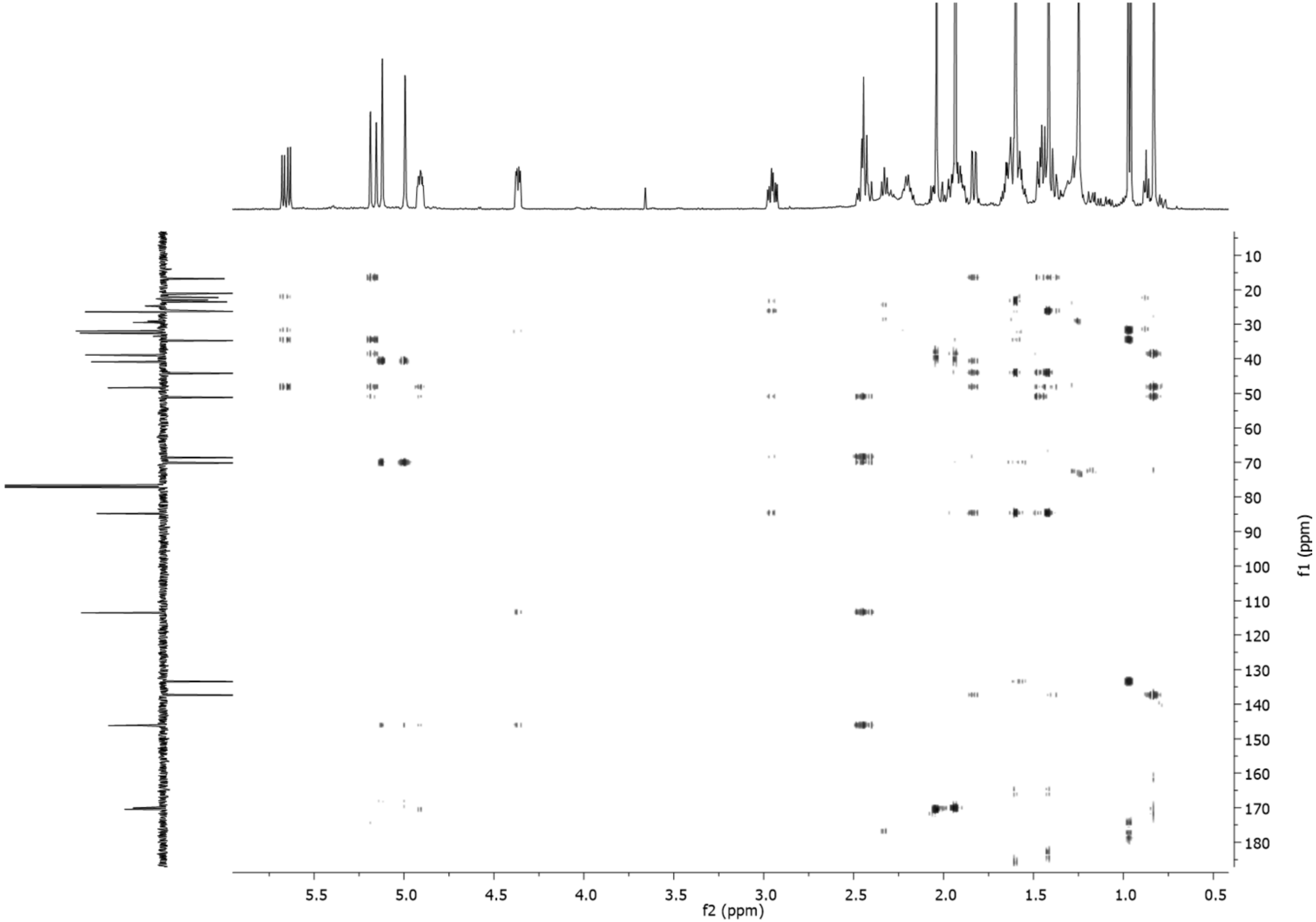

Figure S6. NOESY spectra for compound **1** in $CDCl_3$.

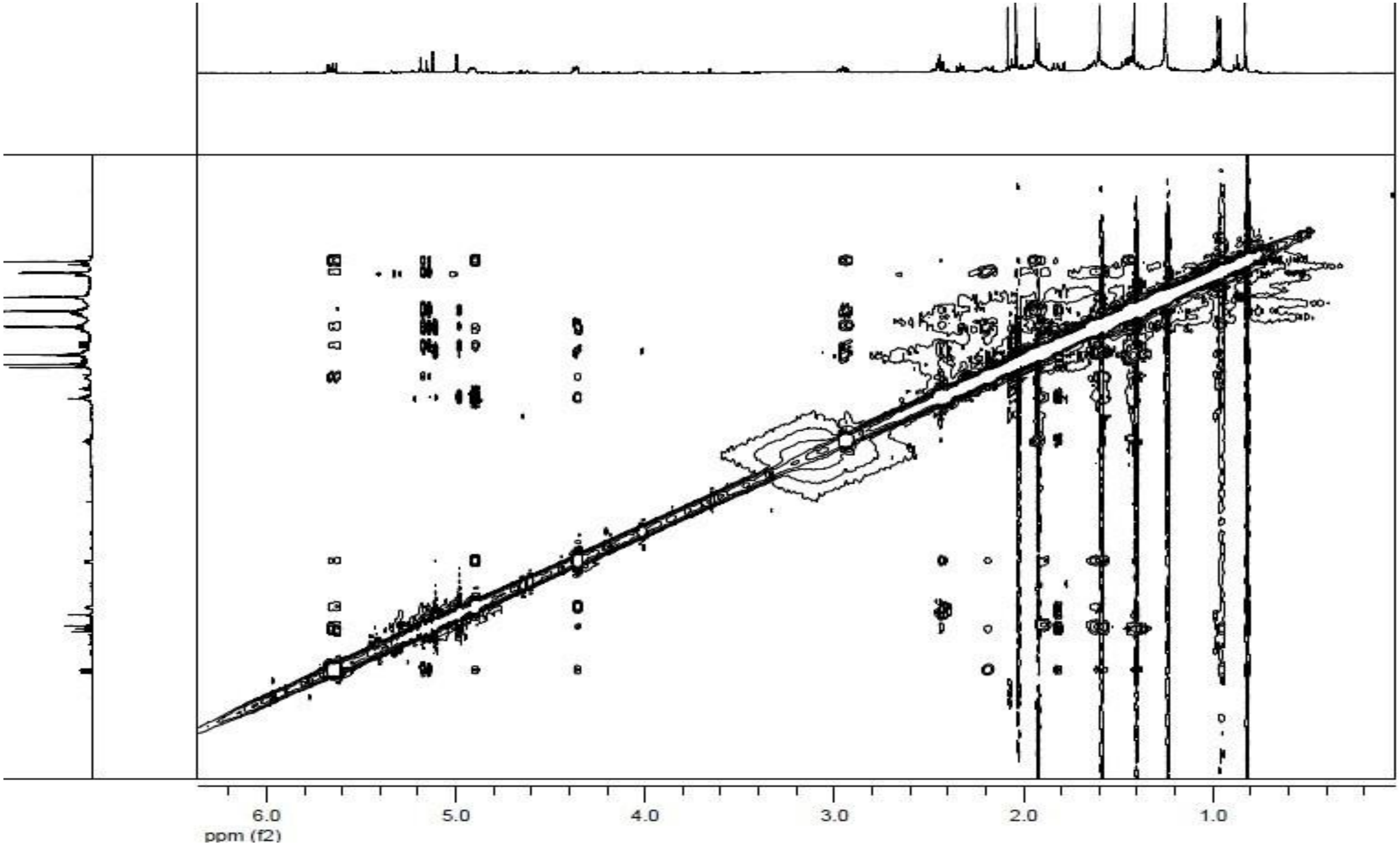

Figure S7. ^{1}H NMR of compound **2** (500 MHz in $CDCl_3$).

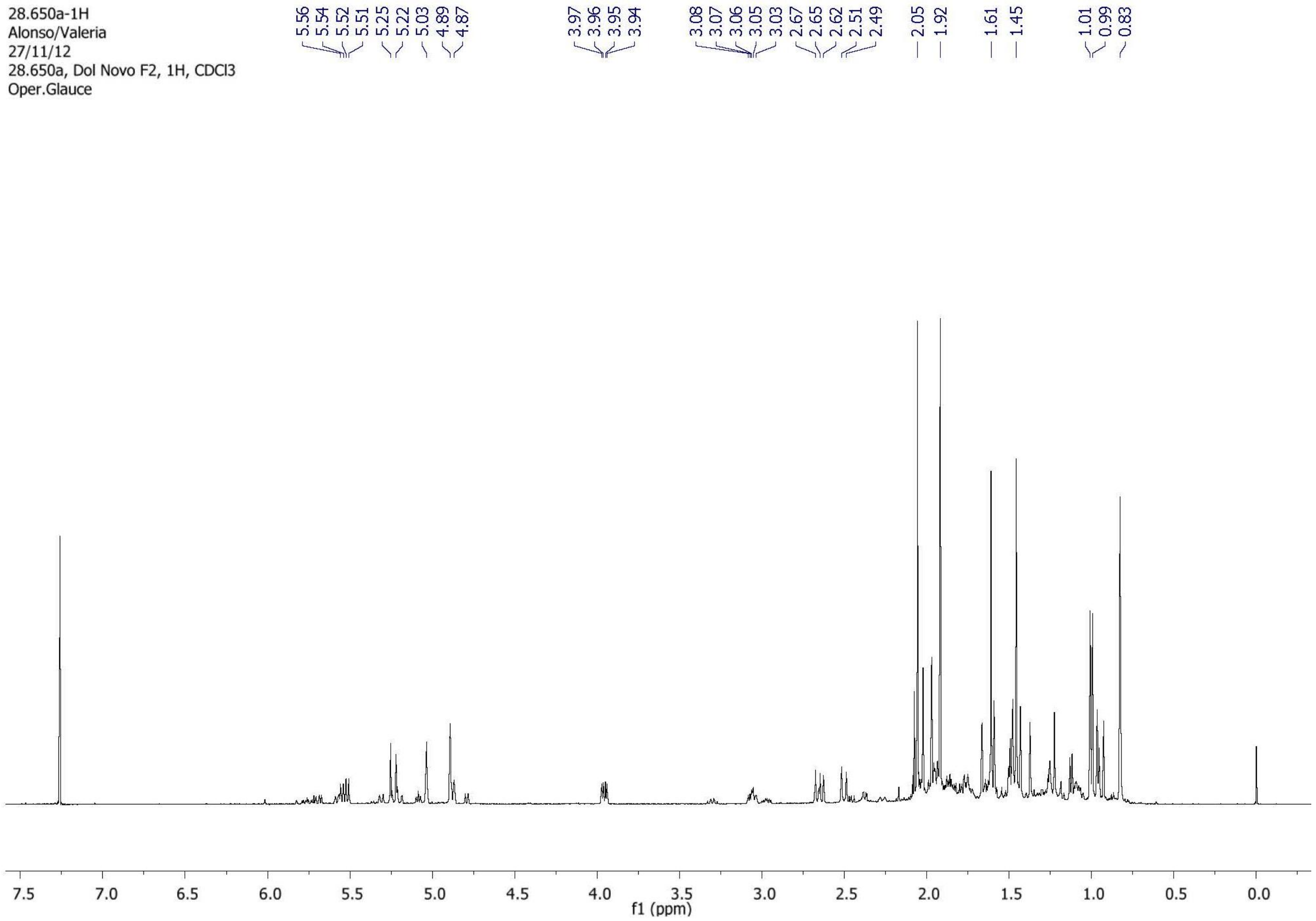

Figure S8. NMR APT spectra for compound **2** (125 MHz in $CDCl_3$).

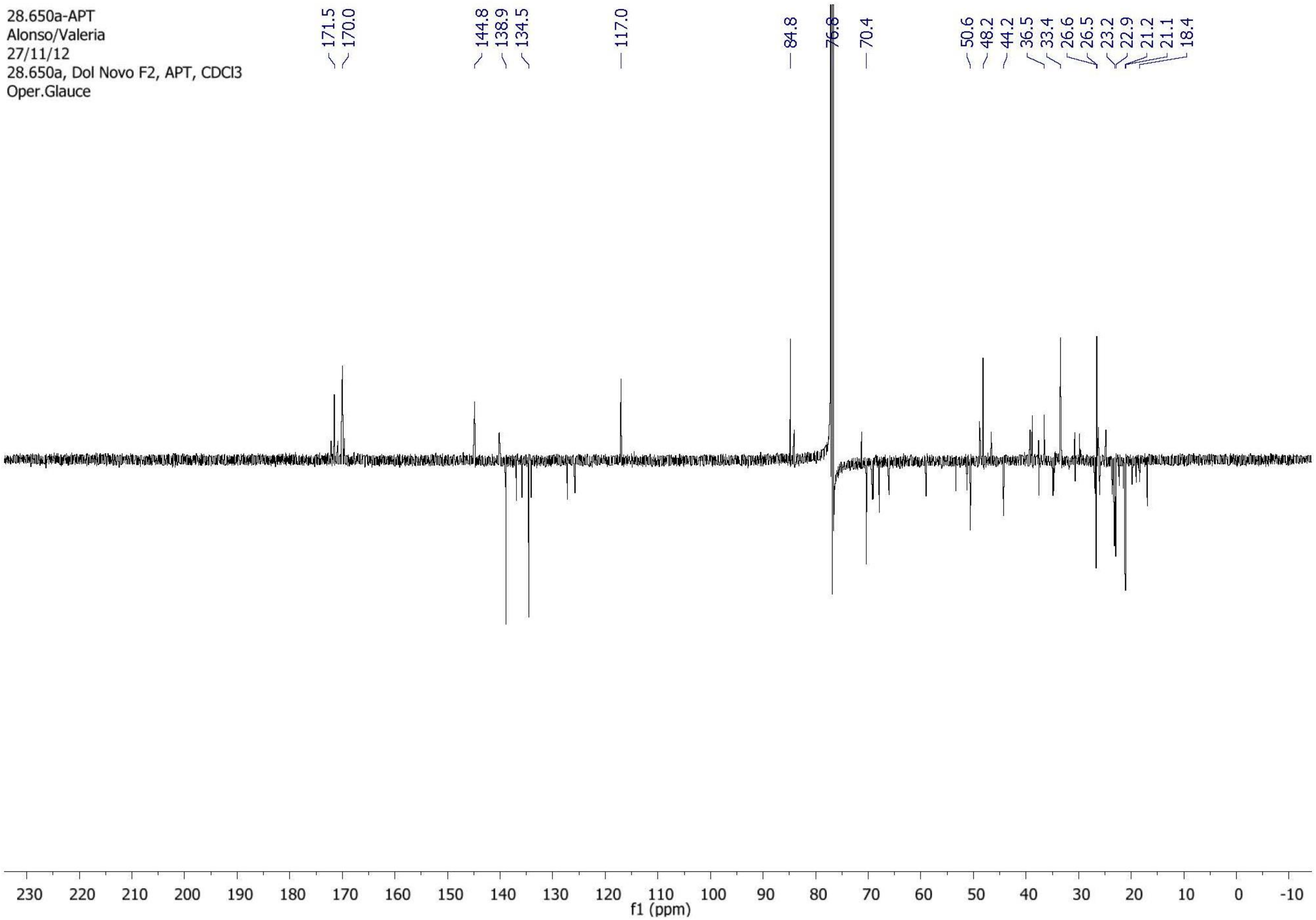

Figure S9. COSY spectra for compound **2** in $CDCl_3$.

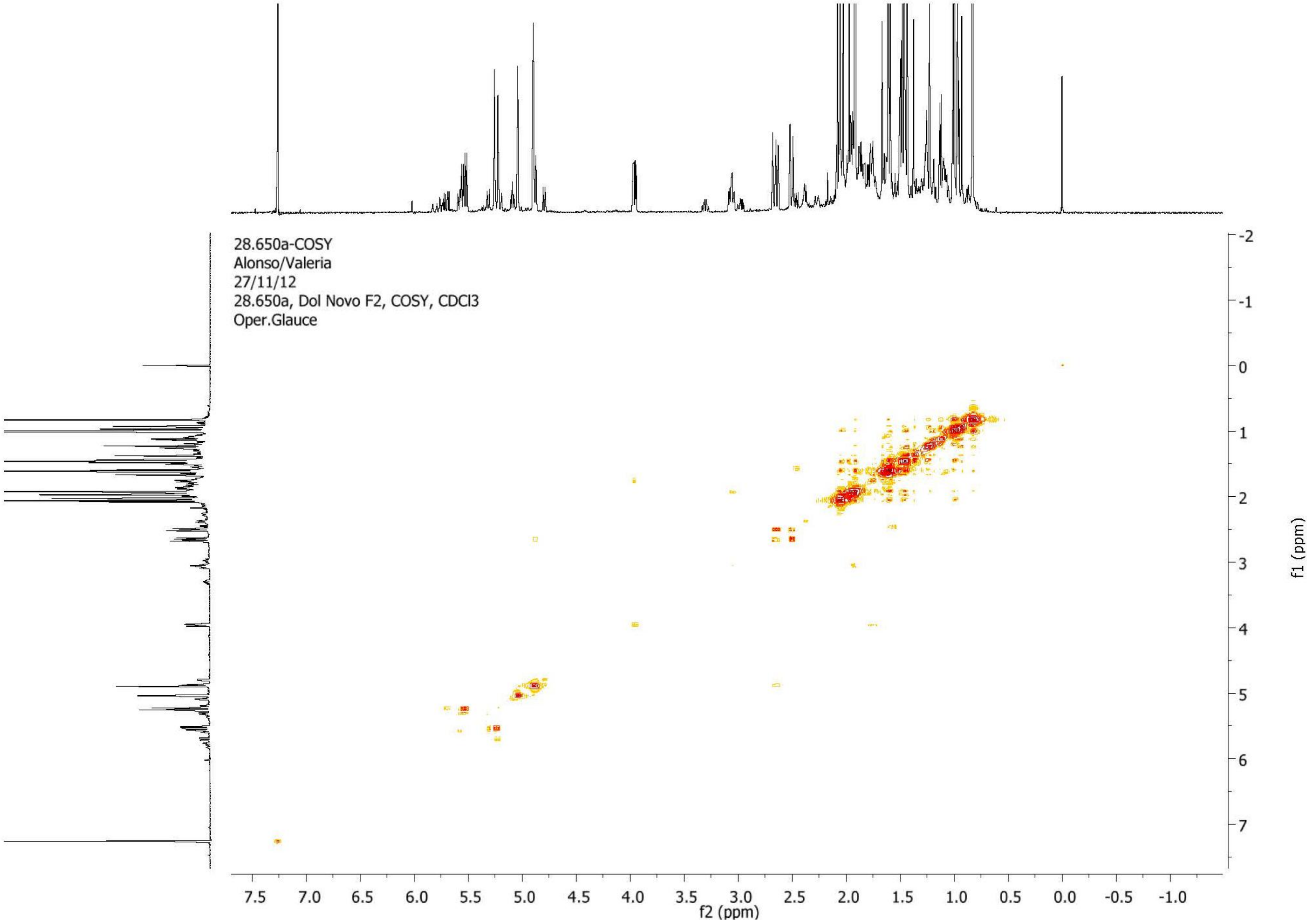

Figure S10. HSQC spectra for compound **2** in $CDCl_3$.

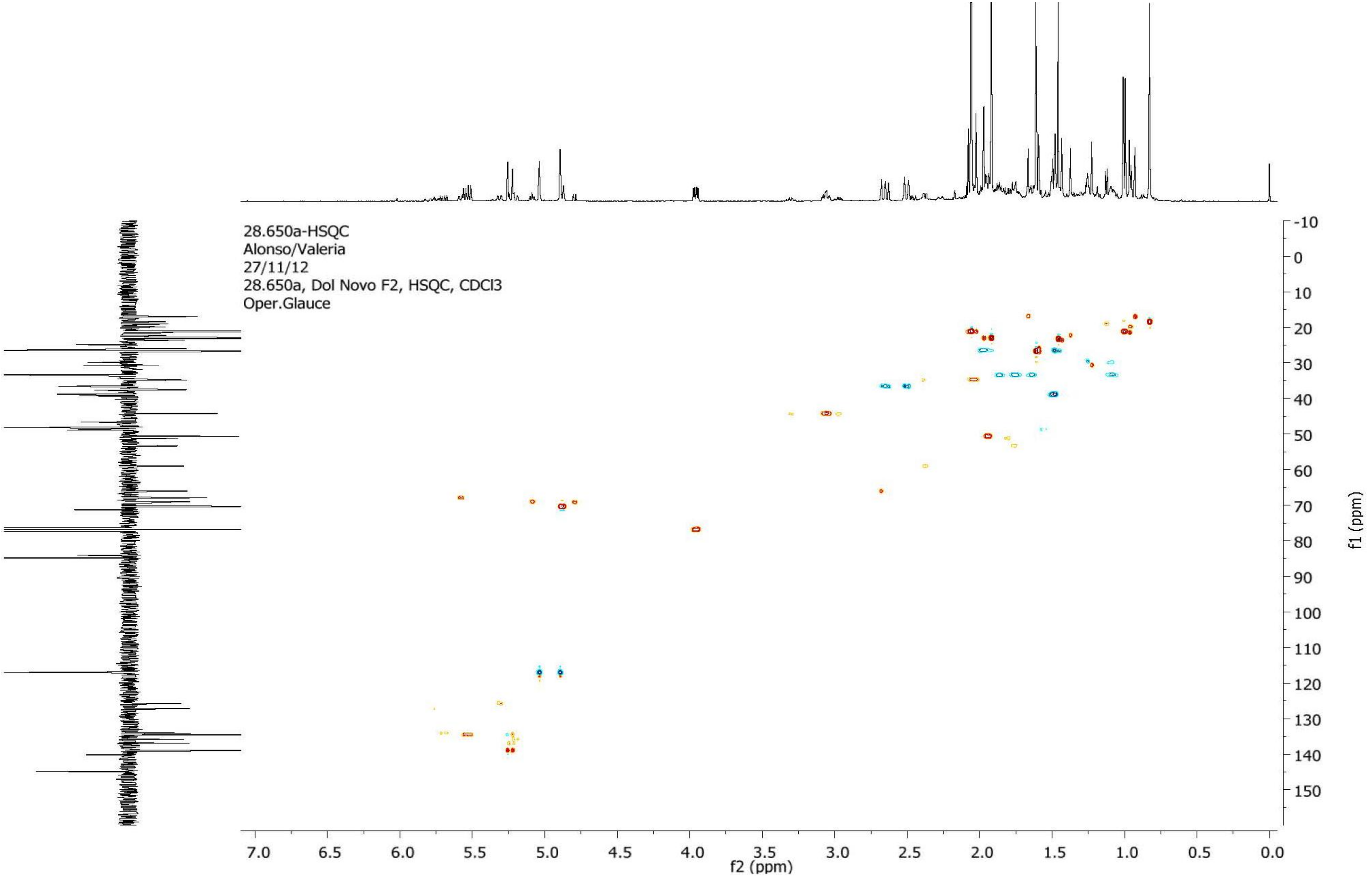

Figure S11. HMBC spectra for compound **2** in $CDCl_3$.

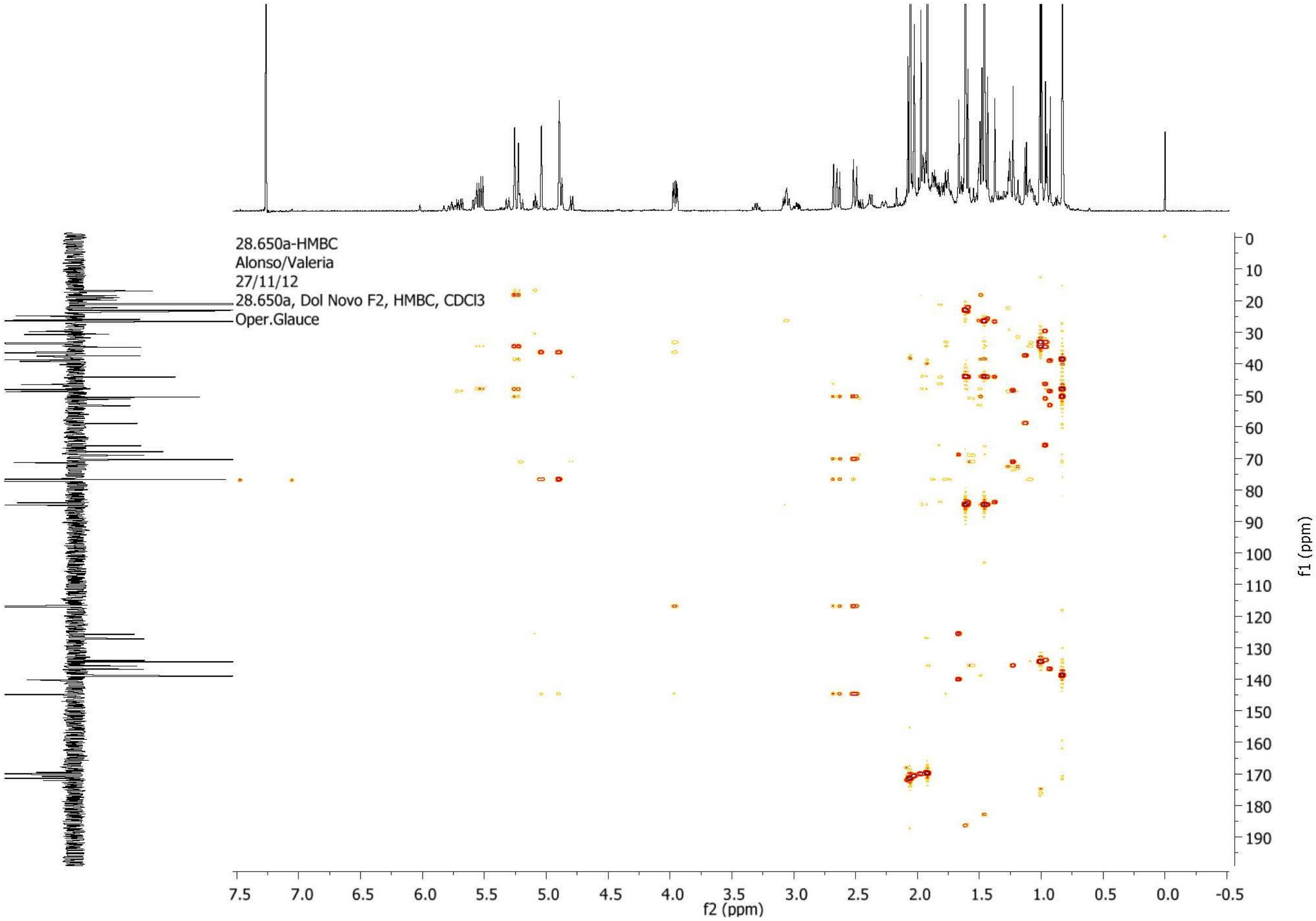

Figure S12. NOESY spectra for compound **2** in $CDCl_3$.

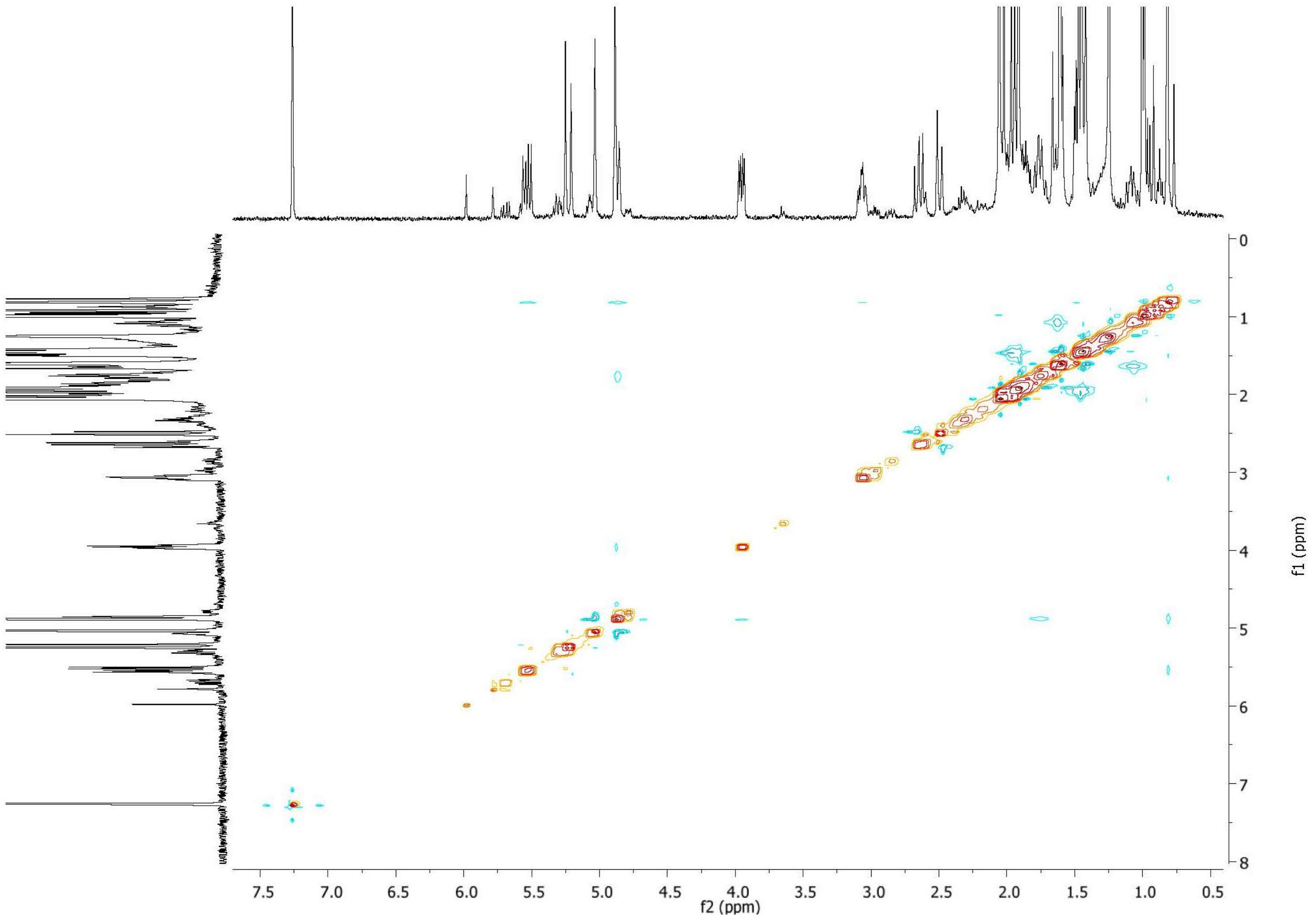

Figure S13. ^{1}H NMR of compound **3** (500 MHz in $CDCl_3$).

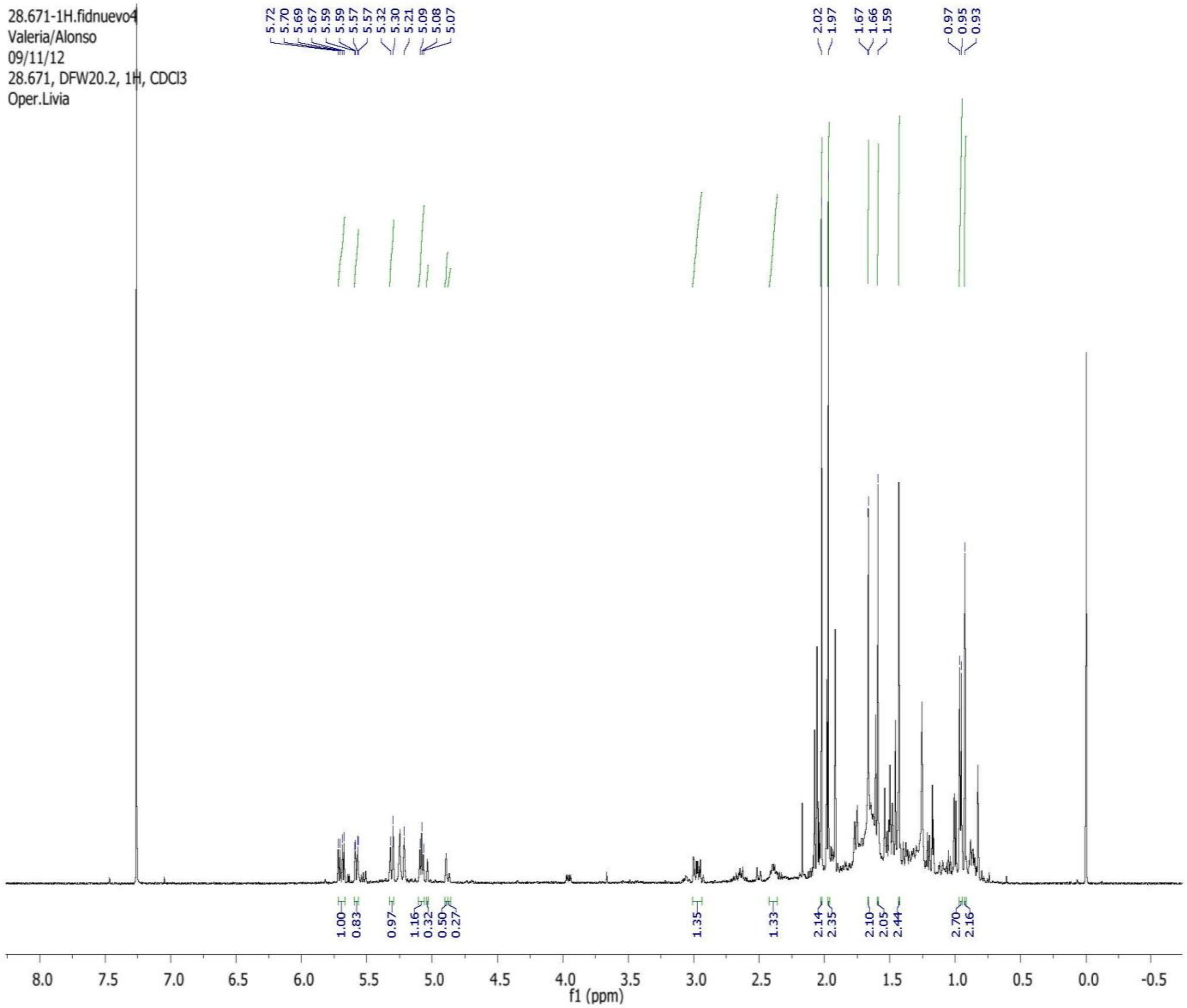

Figure S14. NMR APT spectra for compound **3** (125 MHz in $CDCl_3$).

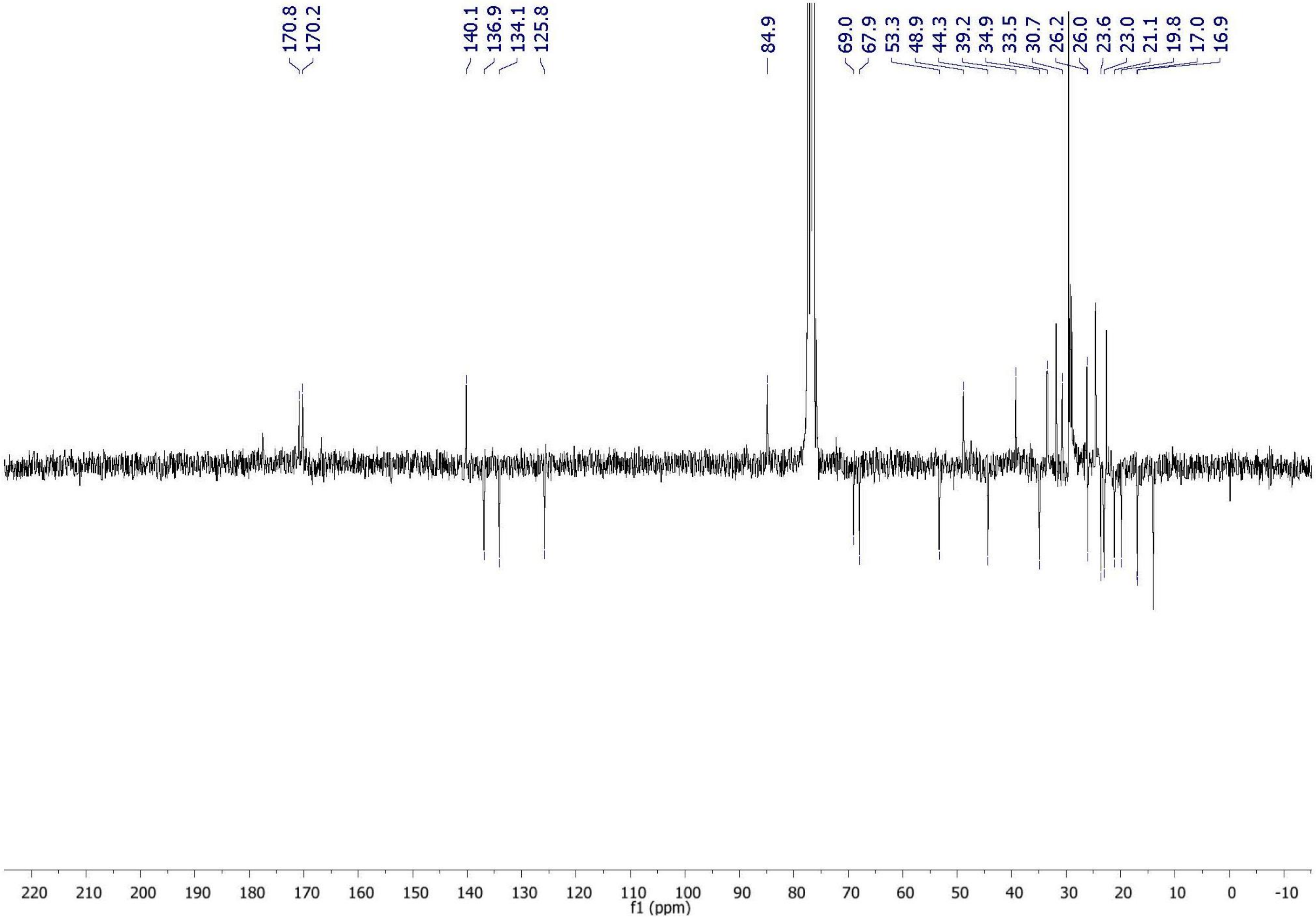

Figure S15. COSY spectra for compound **3** in $CDCl_3$.

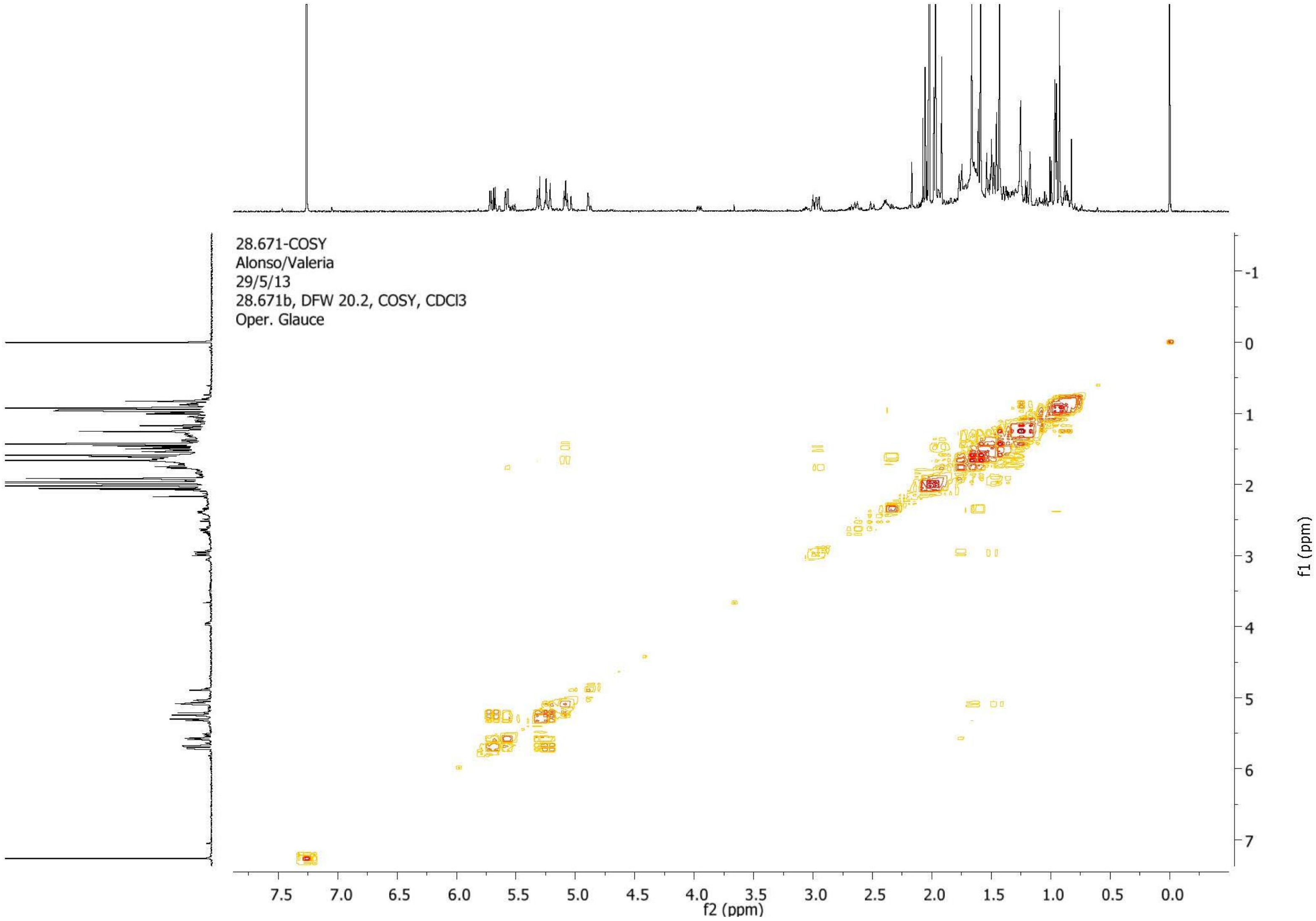

Figure S16. HSQC spectra for compound **3** in $CDCl_3$.

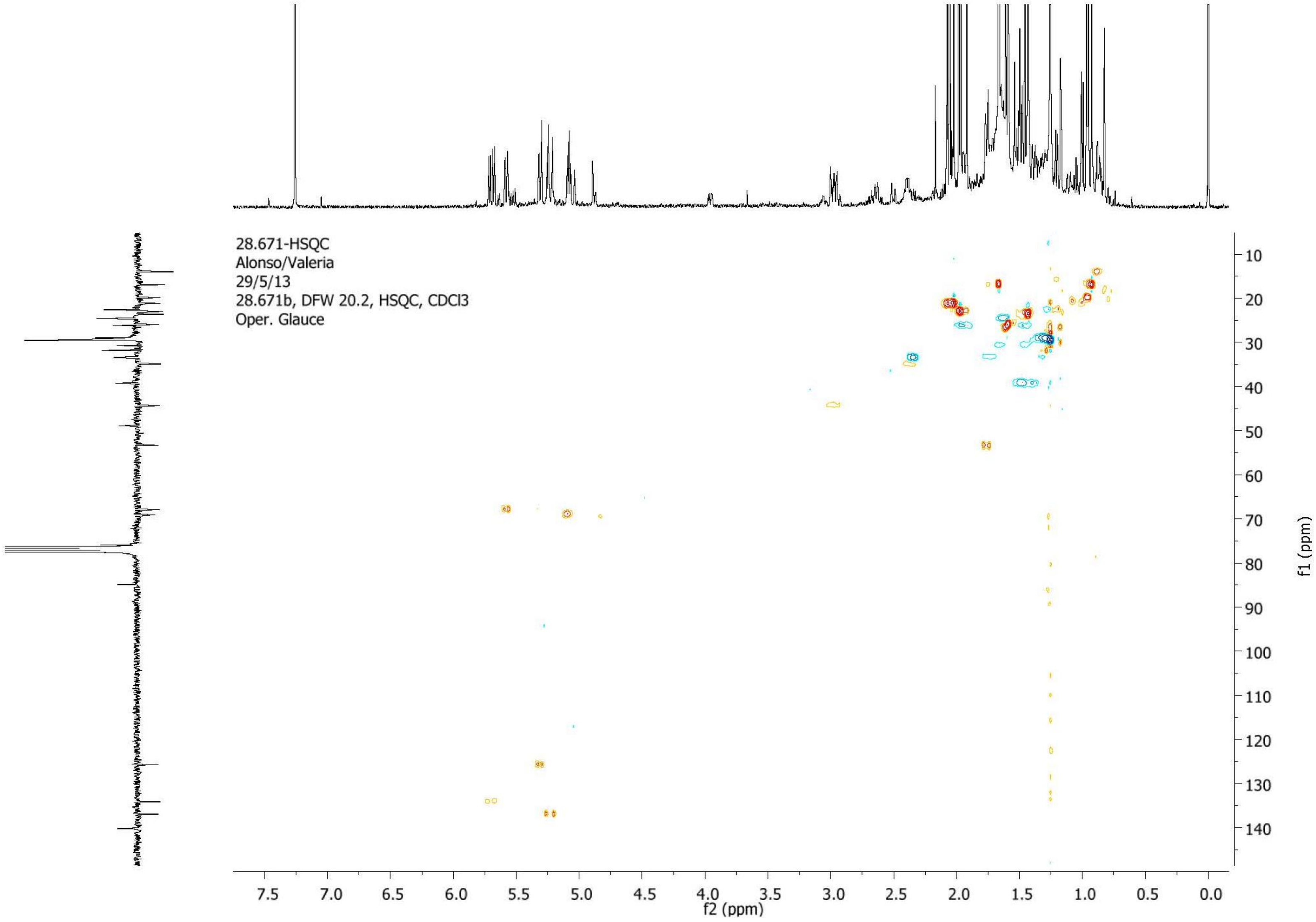

Figure S17. HMBC spectra for compound **3** in $CDCl_3$.

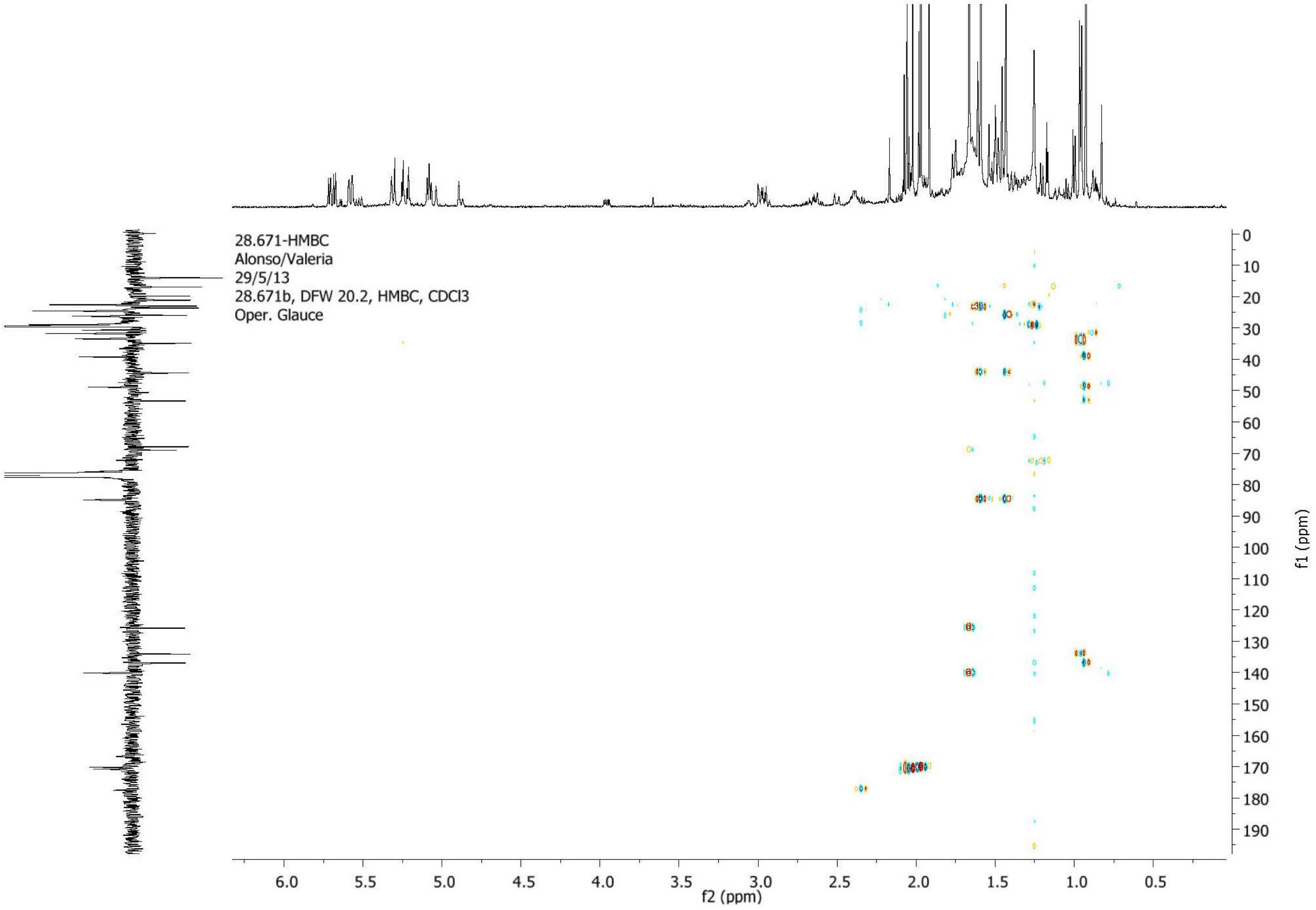

© 2014 by the authors; licensee MDPI, Basel, Switzerland. This article is an open access article distributed under the terms and conditions of the Creative Commons Attribution license (http://creativecommons.org/licenses/by/3.0/).
